# Detection of human parvovirus B19 in papillary thyroid carcinoma

**DOI:** 10.1038/sj.bjc.6604196

**Published:** 2008-01-22

**Authors:** J H Wang, W P Zhang, H X Liu, D Wang, Y F Li, W Q Wang, L Wang, F R He, Z Wang, Q G Yan, L W Chen, G S Huang

**Affiliations:** 1 State Key Laboratory of Cancer Biology, Department of Pathology, Xijing Hospital, Fourth Military Medical University, Xi'an 710032, China; 2 Department of Histopathology, Addenbrooke's Hospital, Cambridge University Hospitals NHS Foundation Trust, Cambridge CB2 2QQ, UK; 3 Department of International Health and Biostatistics, Johns Hopkins Bloomberg School of Public Health, Baltimore, MD 21205, USA

**Keywords:** papillary thyroid carcinoma, human parvovirus B19, virus DNA, viral protein, nuclear factor-*κ*B

## Abstract

To evaluate whether parvovirus B19, a common human pathogen, was also involved in papillary thyroid carcinoma (PTC), 112 paraffin-embedded thyroid specimens of benign nodules, papillary, medullary and follicular carcinomas, and normal controls were examined for B19 DNA and capsid protein by nested PCR, *in situ* hybridisation (ISH) and immunohistochemistry (IHC). The expression of the nuclear factor-*κ*B (NF-*κ*B) was investigated by IHC. The results showed B19 DNA commonly exists in human thyroid tissues; however, there were significant differences between PTC group and normal controls, and between PTC and nonneoplastic adjacent tissues (*P*<0.001). The presence of viral DNA in PTC neoplastic epithelium was confirmed by laser-capture microdissection and sequencing of nested PCR products. B19 capsid protein in PTC group was significantly higher than that of all the control groups and nonneoplastic adjacent tissues (*P*⩽0.001). Compared with control groups, the activation of NF-*κ*B in PTC group was significantly increased (*P*⩽0.02), except for medullary carcinomas, and the activation of NF-*κ*B was correlated with the viral protein presence (*P*=0.002). Moreover, NF-*κ*B was colocalised with B19 DNA in the neoplastic epithelium of PTC by double staining of IHC and ISH. These results indicate for the first time a possible role of B19 in pathogenesis of PTC.

Papillary thyroid carcinoma (PTC) accounts for more than 80 per cent of all the thyroid carcinomas in the United States and other countries (Edwards *et al*, 2002; Mazzaferri and Massoll, 2002). However, very little is known about the aetiology of PTC, although several risk factors such as thyroid irradiation, high dietary iodine intake, autoimmune diseases and genetic alterations have been implicated (Schlumberger, 1998).

Infection with parvovirus B19 (B19) is a global concern, and it is the aetiological agent of many diseases (Bultmann *et al*, 2003). Some researchers have shown that B19 infection may play a role in the pathogenesis of acute leukaemia (Kerr *et al*, 2003). However, little literature on B19 involvement in solid tumours is available.

B19 is a small nonenveloped single-stranded DNA virus with a genome size of 5596 bp. The viral genome encodes three major proteins: the nonstructural protein NS1 and two viral capsid proteins VP1 and VP2 (Young and Brown, 2004). VP1 and VP2 were similar, except for an additional unique portion (VP1u) of 227 amino acids at its N terminus (Figure 1A). NS1 is cytotoxic to host cells (Ozawa *et al*, 1988; Young and Brown, 2004). VP1 and VP2, which form the icosahedral viral capsid, are immunogenic (von Poblotzki *et al*, 1996; Corcoran and Doyle, 2004; Young and Brown, 2004). It is reported that NS1 of B19 resembles tax protein of T-cell leukaemia virus-1 (HTLV-1) and tat protein of human immunodeficiency virus type 1 (HIV) in that they all play a part in viral propagation and activate IL6 production through the Nuclear factor-*κ*B (NF-*κ*B)-binding site in the IL6 promoter (Kerr, 2000). Nuclear factor-*κ*B can be activated rapidly by tax (Hiscott *et al*, 1997) and tat protein (Mhashilkar *et al*, 1997), respectively, and activated NF-*κ*B takes pivotal role in the HTLV-1-induced acute leukaemia (Miwa *et al*, 1997) and HIV-induced Kaposi sarcoma (Nasti *et al*, 1997). The activated NF-*κ*B is also evident in PTC (Bravo *et al*, 2003). We wonder whether B19 protein can activate NF-*κ*B and then plays a role in PTC.

We did a retrospective study to evaluate the possible role of B19 infection in PTC patients. The presence of B19 genome and B19 VP1/VP2 capsid proteins were examined in human thyroid specimens from the consecutive patients with a spectrum of thyroid disorders by nested PCR (nPCR), *in situ* hybridisation (ISH), immunohistochemistry (IHC) and laser-capture microdissection (LCM). The expression of NF-*κ*B and its association with virus infection were investigated by IHC and double labelling of IHC and ISH, respectively. Evidence is presented for a potential role of B19 in the pathogenesis of PTC.

## MATERIALS AND METHODS

### Tissue samples

A total of 112 paraffin-embedded thyroid tissues from the consecutive patients with a spectrum of thyroid disorders were retrieved from the pathology archives of Xijing Hospital from 2000 to 2005. There was no parvovirus epidemic before tissue sampling. We reviewed the patient's charts to ensure that the patients with pre-operative radiation or chemotherapy were excluded and no one was immunocompromised. Two pathologists re-examined all the cases to confirm diagnosis (JHW and GSH). The specimens included 38 PTC tissues (30 out of 38 also had tumour-adjacent nonneoplastic tissues), 16 normal thyroid tissues (obtained from areas surrounding surgically removed adenomas), 39 benign thyroid nodules (20 thyroid adenomas and 19 nontoxic multinodular goiters), 9 thyroid medullary carcinomas (TMC) and 10 follicular thyroid carcinomas (FTC) tissues. Histological classification of tumours was based on the World Health Organization criteria. Kidney tissue of a B19-infected hydropic foetus, analysed earlier by nPCR, ISH and IHC was used as positive tissue control for each experiment. Another foetal kidney tissue of therapeutic abortion without clinical, histological or serological evidence of B19 infection was used as negative tissue control. Specimen collection and the study procedures were approved by the Xijing Hospital Ethics Committee.

### DNA extraction, nested PCR and sequencing

DNA from the paraffin-embedded tissues was isolated by proteinase K digestion followed by phenol–chloroform extraction and ethanol precipitation, and finally, it was resuspended into 200 *μ*l of water.

**Figure 1:**
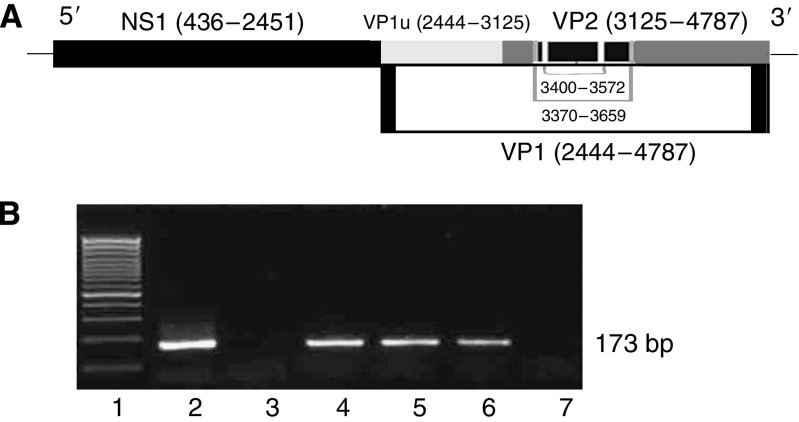
Schematic representation of human parvovirus B19 genome and nested PCR amplification of parvovirus B19 DNA sequences in papillary thyroid carcinomas, (**A**) NS1, VP1, and VP2 coding regions of the B19 genome and the location and nucleotide positions of the primers for nested PCR amplification. (**B**) Representative agarose gel electrophoresis of nested PCR products. Lane 1: 100 bp DNA ladder; lane 2: positive control; lane 3: negative control; lanes 4, 5 and 6: positive samples. The amplicons were approximately 173 bp in size. Lane 7: negative sample.

B19 DNA was amplified by nPCR (Bultmann *et al*, 2003). The first round PCR was carried out with the outer primers (nucleotides 3370–3389 and 3659–3637, respectively). The second round PCR was carried out with the inner primers (nucleotides 3400–3419 and 3572–3552, respectively), which amplified a region of the B19 VP1/VP2 capsid protein sequence (Figure 1A). All the nPCR reactions were performed in duplicate and in parallel with a negative (water) and a positive control (pGEM-1/B19 plasmid carrying the whole genome of B19, kindly provided by Professor JP Clewley of Central Public Health Laboratory, London, UK). The amplified DNA with the expected size (173 bp) was purified with Qiaquick PCR purification kit (Qiagene, Hilden, Germany) and sequenced.

### *In situ* hybridisation

The nPCR products of B19 DNA amplified by TaKaRa Pyrobest DNA polymerase (TaKaRa Biotech) were purified and labelled to generate the B19 DNA probe by random-primed incorporation of digoxigenin-labelled dUTP using a Dig DNA labelling and detection kit (Roche, Mannheim, Germany).

*In situ* hybridisation was performed according to previously described methods (Morey *et al*, 1992). Briefly, paraffin sections of 4 *μ*m thickness were deparaffinised and hydrated, then placed in 0.2 M HCl for 10 min followed by digestion in 25 *μ*g ml^−1^ proteinase K (Merck, Darmstadt, Germany) for 10 min at 37°C. After two washes with PBS, the sections were dehydrated, air-dried and incubated with pre-hybridisation solution at 42°C for 30 min. The sections and the B19 probe (with a final concentration of 200 ng ml^−1^) were simultaneously denatured at 95°C for 10 min, chilled on ice and incubated at 42°C overnight. The slides were then washed sequentially for 15 min twice with SSC containing 10% (w/v) SDS, blocked with blocking buffer at 37°C for 30 min, and incubated for 2 h with anti-digoxigenin-alkaline phosphatase conjugate. After two 15-min washes with washing buffer, the sections were stained with NBT/BCIP (Roche, Mannheim, Germany) solution until satisfactory blue signals were obtained. The sections were then dehydrated and mounted with neutral balsam before cover slipping. The duplicated sections of PTC were hybridised with the pre-hybridisation mixture or digoxigenin-labelled pBR328 DNA linearised with *Bam*HI as negative controls.

### Immunohistochemistry

Paraffin sections of 4 *μ*m thickness were deparaffinised and treated with 3% (v/v) hydrogen peroxide to block endogenous peroxidase activity. Heat-induced antigen retrieval was performed in 0.01 M sodium citrate (pH 6.0) and 10% (v/v) bovine serum albumin (BSA; Sigma, St Louis, MO, USA) in PBS at room temperature for 10 min to block the nonspecific antibody-binding sites. Then the sections were incubated overnight at 4°C with the mouse monoclonal antibody against the B19 proteins VP1/VP2 (1 : 20 dilution; Novocastra, Newcastle, UK) and the mouse monoclonal antibody against the nuclear transcription factor subset NF-*κ*B p65 (1 : 400 dilution; Santa Cruz Biotechnology, Santa Cruz, CA, USA), respectively. A streptavidin–biotin–peroxidase complex kit (SABC kit, Zymed, South San Francisco, CA, USA) was used to detect the protein conjugates (Elias *et al*, 1989). Finally, the sections were developed with a diaminobenzidine substrate (Sigma, St Louis, MO, USA) and counterstained with haematoxylin. Serial sections of PTC were also run in parallel with the replacement of the primary antibody with PBS and mouse IgG1 (AMS/Immunokontact, Abingdon, UK) as negative controls.

### Double staining of immunohistochemistry and *in situ* hybridisation

The expression of NF-*κ*B was marked with mouse anti-NF-*κ*B p65 monoclonal antibody by IHC. Then the section was hybridised with B19-specific DNA probe.

### Laser-capture microdissection and nested PCR

Five positive PTC specimens of B19 confirmed by nPCR were subjected to LCM by the Leica Microsystems Wetzlar GmbH (Wetzlar, Germany), according to the manufacturer's protocols. Serial sections (5 *μ*m) were prepared for each sample. The sections were stained with haematoxylin and eosin. Each section was overlaid with thermoplastic membrane and the cells were captured by focal melting of the membrane by laser activation. Each of the captured samples contained 50–100 cells. The Pico Pure DNA-Extraction kit (Qiagene, Hilden, Germany) was used to isolate DNA from laser-microdissected tissue fractions. Positive and negative controls were included in nPCR. The amplified products with the expected size (173 bp) were purified and sequenced. The individual sequence was then used in a BLAST search against GenBank B19 sequences (National Center for Biotechnology Information). Viral type was identified when nucleotide comparisons revealed an identity of 95% with the known type.

### Statistical analysis

Statistical analysis was performed using Statistical Program for Social Sciences (SPSS) software (version 11.0, SPSS Inc., Chicago, IL, USA). The differences of gender and age distributions among the groups were compared by Pearson *χ*^2^ test and analysis of variance, respectively. Data from the experimental results were analysed by Pearson *χ*^2^ test or Fisher's exact test. Pairwise comparison between PTC and nonneoplastic adjacent tissues was made with McNemar's exact Test. A two-sided *P-*value of <0.05 was considered statistically significant.

## RESULTS

### Clinical aspects and histopathology

The age of the 38 PTC patients ranged from 24 to 75 years old (mean age: 43.5±12.8) and 29 of them were female. Statistical analysis showed there was no significant difference between PTC patients and control groups in age (*P*=0.363) and gender (*P*=0.539). All the PTC patients and control cases were of Chinese origin.

**Figure 2:**
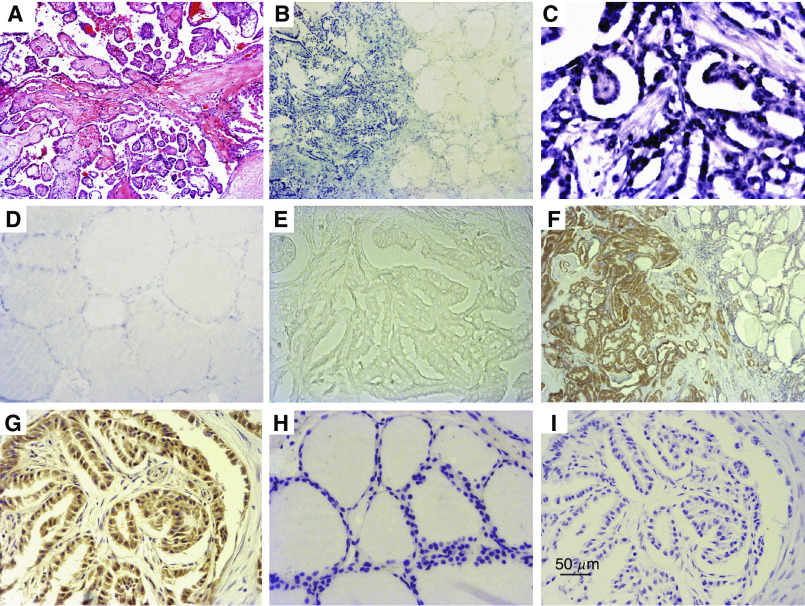
*In situ* hybridization of parvovirus B19 DNA and IHC staining of B19 VP1/VP2 antigen in PTC. (**A**) Typical histopathological features of PTC. (**B** and **C**) The tumour cells of PTC showed strong nuclear staining by ISH, but the staining was almost negative in epithelia in tumour-adjacent tissue (**B**) and normal thyroid tissue (**D**). (**E**) Negative control (B19 probe was substituted by linearized pBR328 DNA). (**F** and **G**) Immunohistochemistry staining was for B19 VP1/VP2 antigen in PTC specimen and positive signals were seen in the cytoplasm of many malignant thyroid epithelia of PTC, but not in tumour-adjacent tissues. (**H**) No expression of VP1/VP2 antigen in normal thyroid samples. (**I**) Negative control (the primary antibody was replaced with mouse IgGI). Original magnification; × 40 (**A**, **B** and **F**); × 200 (**C**–**E**, **G**–**I**).

Histologically, all the PTC specimens included in this study showed typical features of PTC with characteristic papillary structure and/or nuclear features (Figure 2A). All the normal thyroid tissues showed normal morphology. The samples from the patients with nontoxic multinodular goiter were characterised by many nodules, coexisting with atrophic and hyperplastic thyroid follicles, as well as old haemorrhage and fibrosis in stroma. Single nodule with a complete thin fibrous capsule composed of uniform thyroid follicles made the diagnosis of thyroid adenomas. Spindle or round cell tumour with amyloid stroma was the diagnostic feature of TMC. Thyroid nodule containing groups of cells invading the broad collagenous capsule and/or adjacent tissues was the criteria for FTC.

### B19 DNA exists in human thyroid tissues

Using nPCR, a DNA fragment of 173 bp in size as predicted was amplified in 37 out of 38 PTC specimens, 7 out of 16 normal controls, 34 out of 39 benign thyroid nodules, 5 out of 9 TMC and 10 out of 10 FTC specimens (Figure 1B; Tables 1, 2 and 3). DNA sequence analysis performed in four positive samples and the nucleotide comparisons with the known type (GenBank gi: 9632996) revealed similarity of 95–97%, which confirmed that they were amplified from B19 DNA. Statistically, the positive rate of B19 DNA detected by nPCR in PTC group was significantly higher than that in normal controls (*P*=0.0001) and that in TMC (*P*=0.003), respectively.

*In situ* hybridisation for B19 DNA was performed in the tissue sections of PTC and control specimens to confirm the local infection of the virus and its cellular distribution. The positive ISH signals were located in the nuclei of thyroid follicle epithelial cells in different thyroid disorders and they were also seen in isolated or scattered infiltrating lymphocytes. In PTC, the tumour cells showed strong nuclear staining (Figure 2B and C), but no staining or much weak intensity was observed in tumour-adjacent tissue (Figure 2B) or the specimens of normal thyroid (Figure 2D). The negative controls showed no staining (Figure 2E), whereas the positive B19 control sample showed strong staining. The viral DNA was detected in 30 out of 38 PTC, 2 out of 16 normal controls, 24 out of 39 benign thyroid nodules, 1 out of 9 TMC and 7 out of 10 FTC specimens (Tables 1, 2 and 3). The result of ISH was similar to that of nPCR (i.e. the distribution of B19 DNA in the thyroid tissues detected by nPCR was identified by ISH). In addition, there was significant difference between PTC (83.3%, 25 out of 30) and tumour-adjacent tissues (23.3%, 7 out of 30, *P*=0.0001 by McNemar's exact test).

### B19 VP1/VP2-antigen is preferentially located in malignant tissues of PTC

The positive IHC signals of B19 proteins were found in cytoplasm of the follicle epithelial cells in different thyroid disorders. In most PTC specimens, B19 viral protein immunoreactivity was detected in neoplastic epithelium, but it was not seen in tumour-adjacent tissues with normal appearance (Figure 2F and G). Normal epithelium in normal thyroid samples demonstrated no expression of VP1/VP2 antigen (Figure 2H) except only one case was weak positive. The positive B19 control sample gave clear positive staining, whereas the negative controls showed no evidence of IHC staining (Figure 2I). The viral proteins were observed in 24 out of 38 PTC specimens, 1 out of 16 normal control thyroid tissues and 8 out of 39 benign thyroid nodules, but no viral protein was found in TMC and FTC. Statistically, the positive rate of B19 VP1/VP2 antigen in PTC group was significantly higher than that in the other groups (all *P*⩽0.001). Pairwise comparisons showed a significant difference in VP1/VP2 immunoreactivity between PTC (63.3%, 19 out of 30) and tumour-adjacent tissues (10.0%, 3 out of 30, *P*=0.0001 by McNemar's exact test).

### The activation of NF-*κ*B is correlated with the VP1/VP2-antigen expression

**Figure 3:**
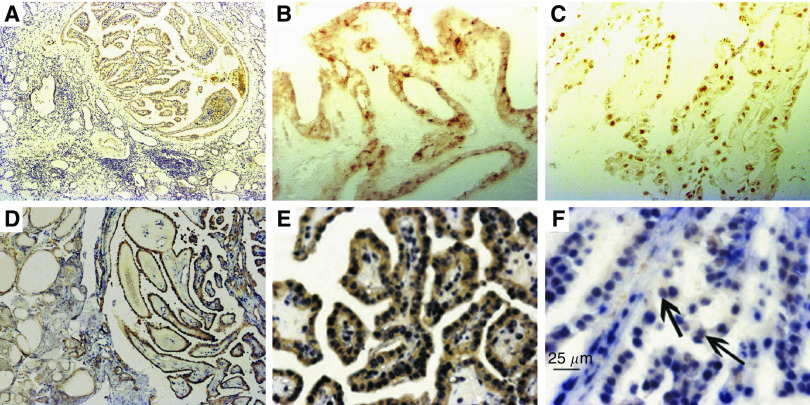
Immunohistochemical staining of NF-*κ*B p65 and double labelling of NF-*κ*B p65 and B19 DNA in the samples of PTC. (**A**) NF-*κ*B immunoreactivity in malignant tissues of PTC, but not in tumour-adjacent tissues. (**B**) Immunoreactivity of NF-*κ*B in the cytoplasma of a PTC sample. (**C**) Nuclear translocation of NF-*κ*B in a PTC sample. (**D**) NF-*κ*B IHC and B19 DNA ISH double staining signal in malignant epithelium in PTC, but the staining intensity was much weak in epithelia in tumour-adjacent tissue. (**E**) Cytoplasmal expression of NF-*κ*B p65 protein and robust nuclear presence of B19 DNA in identical tumour cells. (**F**) Coexpression of NF-κB p65 protein and B19 DNA in the nuclei of the tumour cells of PTC (black arrow). Original magnification: × 40 (**A** and **D**); × 200 (**B**, **C** and **E**); × 400 (**F**).

The positive signal of NF-*κ*B in cytoplasm (Figure 3A and B) was detected in 29 out of 38 PTC specimens by IHC. The expression of NF-*κ*B was greatly increased when compared with normal (2 out of 14, *P*=0.0001), benign control groups (18 out of 37, *P*=0.013), FTC (1 out of 10, *P*=0.0001) and tumour-adjacent tissue (1 out of 30, *P*=0.0001). Nuclear translocation of NF-*κ*B was found in 16 out of 38 PTC (Figure 3C), whereas only in 3 out of 37 benign control group (*P*=0.001) and 1 out of 30 tumour-adjacent tissue (*P*=0.0001). None was observed in 14 normal controls (*P*=0.002) and 10 FTC (*P*=0.02). There was no significant difference between PTC and TMC in both cytoplasm (4 out of 9, *P*=0.102) and nuclear expression (4 out of 9, *P*=1.000) of NF-*κ*B.

In 33 cases of thyroid tissues that were positive for B19 VP1/VP2 protein, nuclear translocation of NF-*κ*B was found in 13 cases, whereas in 75 cases that were negative for VP1/VP2 protein, no staining or immunostaining is present only in the cytoplasm in 65 cases. The nuclear translocation of NF-*κ*B was well correlated with the VP1/VP2-antigen expression (13 out of 33, *P*=0.002).

The IHC-ISH double staining assay demonstrated the expression of NF-*κ*B p65 was colocalised with B19 DNA in cytoplasm (Figure 3D and E) or nuclei (Figure 3F) in the neoplastic cells of PTC.

### The presence of viral DNA in PTC neoplastic epithelium was confirmed by LCM and sequencing of nested PCR products

**Figure 4:**
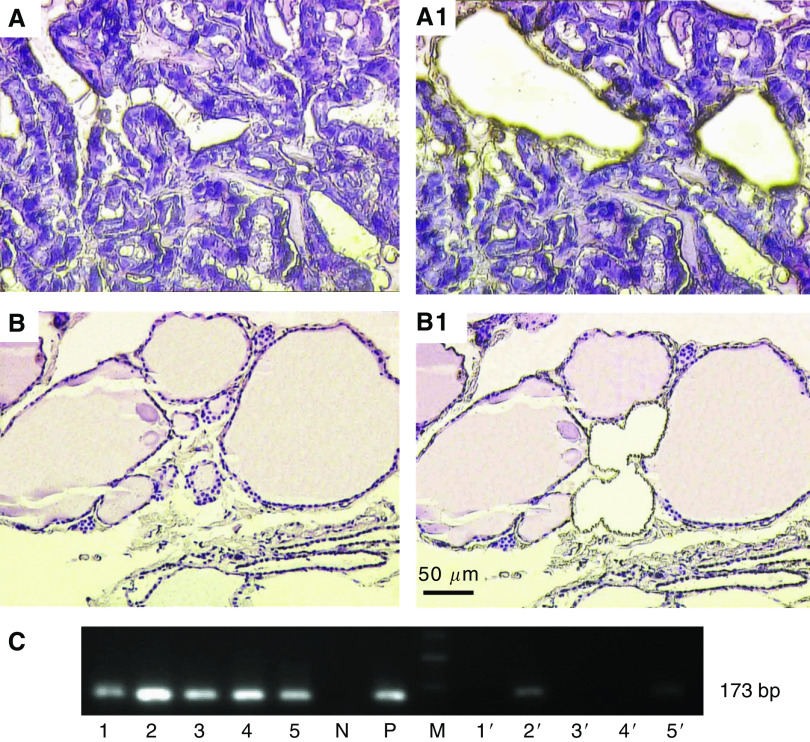
Laser-capture microdissection and nested PCR amplification for parvovirus B19 DNA in papillary thyroid carcinoma. (**A**–**A1**) Tumour cells of papillary thyroid carcinoma were microdissected. (**B**–**B1**) Glandular structures of tumour-adjacent tissue of PTC were microdissected. (**C**) Products of nested PCR from the microdissected tissues. Lane M: 100 bp DNA ladder. Lanes 1–5: specimens from neoplastic tissues; lanes 1′–5′: tumour-adjacent tissues; lane N: negative control; lane P: positive control. The amplicons were approximately 173 bp in size. Original magnification: × 400 (**A**–**A1**); × 200 (**B**–**B1**).

Laser-capture microdissection was performed in two different regions of five PTC samples. In the first region, PTC tumour cells were dissected (Figure 4A1 and A2) and in the second region glandular structures of tumour-adjacent tissues were dissected (Figure 4B1 and B2). B19 genome was detected in all the microdissected PTC sections by nested PCR, while only two were detected in matched tumour-adjacent tissues (Figure 4C). Sequencing analysis of the PCR amplicons showed that they were from human parvovirus B19 gene.

## DISCUSSION

Although nPCR was reported to be the most sensitive method, PCR-based method alone for viral detection was not sufficient (Pagano *et al*, 2004). In addition, PTC contains hypervascularised fibrous connective tissue core which could interfere with the nPCR results. Therefore, ISH together with IHC were performed to confirm the local infection of the virus and its cellular distribution, and the presence of viral DNA in PTC neoplastic epithelium was confirmed by laser-capture microdissection and sequencing of nested PCR products. Combination of these methods allowed us to acquire definite histopathological information on B19 infection while maintaining a high degree of sensitivity.

In the present study, both B19 genome and the virus capsid proteins were frequently found in PTC tissues compared with normal controls and nonneoplastic adjacent tissues. Meanwhile, NF-*κ*B was found to be activated, and it was well colocalised with the virus DNA in PTC tissues. Our findings revealed for the first time that there is a novel link between B19 and PTC.

From our study, we found that the positive rate of B19 DNA was high in benign thyroid nodules, FTC and PTC but low in normal specimens. The different positive rates of B19 DNA could be due to the preference for the rapidly dividing cells in S phase for virus replication (Wolter *et al*, 1980). It is reported that the proliferative activity of thyroid adenomas, nontoxic multinodular goiters, follicular thyroid carcinoma and PTC is higher than that in normal thyroid tissue (Saiz *et al*, 2002). Therefore, it is comprehensible that B19 infection rates in these groups are higher than that in normal thyroid tissue in this study.

Several members of the parvovirinae family, including H-1 virus and minute virus of mice, have been shown to prevent tumour formation by their oncotropism and oncolysis in laboratory animals (Rommelaere and Cornelis, 1991). If B19 has oncotropic and oncolytic properties, cell death should be obvious in PTC. However, morphologically, necrosis is not a feature in PTC. On the contrary, PTC often shows high proliferative activity (Saiz *et al*, 2002) and very low apoptotic index (Basolo *et al*, 1997). Therefore, whether there is another explanation for the high frequency of B19 in PTC is in question.

In our study, we also found B19 DNA in TMC is less frequent than that in the other thyroid diseases. This phenomenon could result from the specific cell tropism of the B19 (Brown *et al*, 1993). It is well known that B19 can only infect the cells that have the proper receptors to which the virus can attach. Human blood group P antigen has been confirmed to be the indispensable cellular receptor for B19 infection (Brown *et al*, 1994). Human thyroid follicle epithelia have been shown to have globoside (Bouchon *et al*, 1985). Therefore, the expression of the specific cellular receptor of B19 in these cells might account for their susceptibility to B19 infection. Thyroid medullary carcinomas originated from C cells, the low positive rate of B19 DNA in TMC might be due to the absence of the specific cell receptor for B19 infection. Further works are required to know the possible reason.

It is in recent years that Norja *et al* (2006) have found that the persistence of B19 genome in human tissues is ubiquitous and lifelong. Another recent publication has demonstrated short regions of sequence identity between B19 and human genes, and these sequence identity may be biologically relevant to the persistence of the viruses in human tissues (Kerr and Boschetti, 2006). Biopsies of tissues from synovium, skin, tonsils and liver showed the presence of B19 genome; however, whether the virus genome persists in thyroid tissue has not been determined. Furthermore, without the expression of virus protein, the persistence of B19 genome mainly denotes latent infection. Usually, the detection of the persistence of viral genome with expressed viral proteins in the tumour cells indicates a productive infection of the virus in the cells and the potential involvement of virus in pathways leading to the development and/or progression of cancer (Pagano *et al*, 2004). In our study, we found that B19 DNA existed in 37 out of 38 PTC specimens and VP1/VP2-antigen was preferentially located in tumour tissues of PTC, which indicated that the virus was active in PTC lesions and the productive infection of the virus might play some role, directly or indirectly, as a cofactor in the pathogenesis of the tumour. In addition, as benign thyroid proliferative disease may become neoplastic (Pang *et al*, 1994; Bravo *et al*, 2003; Moore, 2006) and some benign thyroid diseases without histopathological evidence of PTC harbour similar molecular genetic changes with PTC (Wirtschafter *et al*, 1997; Rhoden *et al*, 2006), the detection of B19 VP1/VP2 antigen in 6.25% of normal controls, 10% of tumour-adjacent tissues, 20.5% of benign thyroid tissues and 63.2% of PTC samples suggests that B19 expression may occur relatively early in carcinogenesis of PTC.

Parvovirus is tightly dependent on host functions to complete their life cycle (Wolter *et al*, 1980). Once the appropriate cellular conditions are met, the virus starts its replication at the G1/S transition, and a lytic or even productive infection can ensue. Completion of the viral life cycle requires the assistance of various cellular molecules (Raab *et al*, 2002); some of these have been identified, however, much is unknown. In our study, all samples of FTC were B19 negative by IHC, whereas they showed comparable rates of positivity by PCR and ISH, which indicated that the virus can infect thyroid epithelial cell but was active only in PTC lesions. Papillary thyroid carcinoma and FTC are both carcinomas of follicular cell origin; however, distinct susceptibility factors have been involved in the pathogenesis of them. Presumably, some early changes in PTC patients that promote parvovirus replication did not exist in FTC. That is to say the different early changes in FTC are unlikely to fulfill the requirements for B19 virus replication and transcription.

Although the pathogenesis of PTC is not fully understood, it is known that activation of NF-*κ*B, a mediator for viral-induced tumorigenesis (Mosialos, 1997), plays a critical role in the process of thyroid cell transformation (Visconti *et al*, 1997). In our study, we found the NF-*κ*B was overexpressed and activated in PTC, and the activation of NF-*κ*B was correlated with the expression of viral protein (13 out of 33, *P*=0.002). These results support the hypothesis that B19 may play a role in the pathogenesis of PTC; however, further work is required to address the association between NF-*κ*B and B19 protein, and between B19 and PTC. The present results showed TMC also had a substantial percentage of NF-*κ*B nuclear expression. This phenomenon is in agreement with prior studies in medullary carcinomas (Ludwig *et al*, 2001). A variety of stimuli can initiate different signalling pathways leading to NF-*κ*B activation, and specific point mutations of the *RET* proto-oncogene in TMC is responsible for NF-*κ*B activation and *RET*-mediated carcinogenesis.

Indeed, determining the relationship between a virus infection and a cancer is not an easy task. Based on our findings that both B19 nucleic acids and proteins are in a significantly high proportion in malignant tissues of PTC, we believe that there is some correlation between parvovirus B19 and PTC. However, further work is required to investigate the exact role of B19 infection in PTC.

## Figures and Tables

**Table 1:** PCR, *in situ* hybridization and immunohistochemical results for paraffin specimens of human papillary thyroid carcinoma and nonneoplastic-adjacent tissues

								**IHC of NF-*κ*B p65**			
				**ISH**		**IHC**		**Cytoplasmic**		**Nuclear**	
**Case**	**Age, years**	**Sex**	**nPCR**	**Tumour**	**Adjacent-tumour**	**Tumour**	**Adjacent-tumour**	**Tumour**	**Adjacent-tumour**	**Tumour**	**Adjacent-tumour**
1	36	M	+	+	−	−	−	+	−	+	−
2	34	F	+	−	−	−	−	−	−	−	−
3	62	F	+	+	+	+	+	+	+	−	−
4	61	M	+	+	−	−	−	+	−	−	−
5	39	F	+	+	NT	+	NT	+	−	−	−
6	41	F	+	+	+	+	+	+	−	−	−
7	47	F	+	+	+	−	−	−	−	−	−
8	59	M	+	+	+	+	+	−	−	+	−
9	28	F	+	+	−	+	−	−	−	−	−
10	28	F	+	+	−	−	−	−	−	−	−
11	49	F	+	−	−	−	−	−	−	+	+
12	24	F	+	+	+	−	−	+	−	+	−
13	33	F	+	+	−	−	−	+	−	−	−
14	40	F	+	+	−	+	−	+	−	+	−
15	62	F	+	+	NT	+	NT	+	−	+	−
16	39	F	+	−	NT	−	NT	−	−	−	−
17	64	M	+	−	NT	−	NT	+	−	+	−
18	37	F	+	+	NT	+	NT	−	−	−	−
19	60	M	+	+	−	+	−	+	−	+	−
20	48	F	+	+	−	+	−	+	−	+	−
21	30	M	+	+	NT	+	NT	+	−	−	−
22	68	M	+	+	NT	+	NT	+	−	+	−
23	30	F	+	−	NT	−	NT	+	−	−	−
24	75	F	+	+	−	+	−	+	−	+	−
25	35	F	+	+	−	+	−	+	−	−	−
26	29	F	+	+	−	+	−	+	−	−	−
27	33	F	+	+	−	+	−	+	−	+	−
28	34	F	+	+	−	+	−	+	−	−	−
29	47	F	+	−	−	−	−	+	−	−	−
30	42	F	+	+	−	+	−	+	−	+	−
31	44	F	+	+	−	+	−	+	−	+	−
32	33	F	+	+	+	+	−	+	−	+	−
33	38	M	+	+	−	+	−	+	−	−	−
34	37	F	+	+	+	+	−	+	−	−	−
35	42	F	+	+	−	+	−	+	−	−	−
36	41	F	−	−	−	−	−	+	−	−	−
37	47	F	+	−	−	−	−	−	−	−	−
38	57	M	+	+	−	+	−	+	−	+	−

IHC=immunohistochemistry; ISH=*in situ* hybridisation; NF-*κ*B=nuclear factor kappa B; nPCR=nested PCR; NT=no nonneoplastic-adjacent tissues.

**Table 2:** PCR, *in situ* hybridization and immunohistochemical results for paraffin specimens of human control thyroid tissues

			**B19 status**			**IHC of NF-*κ*B p65**	
**Case**	**Age, years**	**Sex**	**nPCR**	**ISH**	**IHC**	**Cytoplasmic**	**Nuclear**
*Normal controls*							
1	36	F	+	−	−	−	−
2	42	F	−	−	−	+	−
3	67	F	−	−	−	−	−
4	59	F	+	−	−	ND	ND
5	44	F	+	+	−	ND	ND
6	25	F	−	−	−	−	−
7	57	F	+	+	+	+	−
8	49	F	−	−	−	−	−
9	60	M	−	−	−	−	−
10	45	F	+	−	−	−	−
11	58	F	+	−	−	−	−
12	36	F	−	−	−	−	−
13	51	F	−	−	−	−	−
14	40	F	+	−	−	−	−
15	32	F	−	−	−	−	−
16	48	F	−	−	−	−	−
							
*Benign thyroid nodules*							
1	41	F	+	+	+	+	−
2	52	F	+	+	+	−	−
3	52	F	+	+	−	+	−
4	28	F	+	−	−	−	−
5	56	M	+	−	−	−	−
6	41	M	+	−	−	−	−
7	22	F	+	−	−	−	−
8	59	M	+	−	−	+	−
9	21	F	+	+	−	−	−
10	58	F	+	+	+	−	−
11	54	F	−	−	−	−	−
12	43	F	−	−	−	ND	ND
13	39	F	−	−	−	−	−
14	38	F	+	+	−	−	−
15	42	F	+	+	+	+	−
16	34	F	+	−	−	−	−
17	51	F	+	−	−	+	−
18	68	F	+	+	+	+	−
19	41	F	+	+	−	−	−
20	25	F	+	+	+	+	−
21	28	F	+	+	−	+	+
22	40	F	+	+	−	−	−
23	53	F	+	+	+	+	+
24	50	F	+	+	−	+	−
25	35	F	+	+	−	+	−
26	51	M	−	−	−	+	−
27	44	M	+	+	−	+	+
28	62	M	+	+	+	+	−
29	21	F	−	−	−	−	−
30	39	F	+	+	−	ND	ND
31	54	F	+	+	−	+	−
32	57	F	+	−	−	−	−
33	54	F	+	+	−	+	−
34	41	F	+	−	−	−	−
35	46	F	+	+	−	+	−
36	48	F	+	+	−	−	−
37	51	F	+	−	−	−	−
38	32	F	+	+	−	−	−
39	63	F	+	+	−	+	−
							
*Medullary thyroid carcinoma*							
1	33	F	−	−	−	+	+
2	68	F	−	−	−	−	−
3	68	F	+	+	−	+	+
4	55	M	+	−	−	+	+
5	30	F	+	−	−	−	−
6	39	F	−	−	−	−	−
7	58	M	−	−	−	+	+
8	46	F	+	−	−	−	−
9	32	F	+	−	−	−	−
*Follicular thyroid carcinoma*							
1	37	F	+	+	−	−	−
2	72	F	+	+	−	+	−
3	35	M	+	−	−	−	−
4	57	F	+	−	−	−	−
5	56	F	+	+	−	−	−
6	33	F	+	+	−	−	−
7	64	F	+	+	−	−	−
8	57	F	+	+	−	−	−
9	41	F	+	+	−	−	−
10	70	F	+	−	−	−	−

IHC=immunohistochemistry; ISH=*in situ* hybridisation; ND =not done as the blocks were used up; NF-*κ*B=nuclear factor kappa B; nPCR=nested PCR.

**Table 3:** Positive rate (%) of parvovirus B19 detected by nPCR, ISH and IHC in thyroid tissues of PTC and control groups

**Group**	**A**	**B**	**C**	**D**	**E**	***P*-value^a^**			
**Disease**	**PTC (*n*=38)**	**Normal controls (*n*=16)**	**Benign nodules (*n*=39)**	**TMC (*n*=9)**	**FTC (*n*=10)**	**A *vs* B**	**A *vs* C**	**A *vs* D**	**A *vs* E**
nPCR	97.4	43.8	87.2	55.6	100.0	0.0001	0.200	0.003	1.000
ISH	78.9	12.5	61.5	11.1	70.0	0.0001	0.095	0.0001	0.657
IHC	63.2	6.25	20.5	0.0	0.0	0.0001	0.0001	0.001	0.0001

FTC=follicular thyroid carcinoma; IHC=immunohistochemistry; ISH=*in situ* hybridization; nPCR=nested PCR; TMC=thyroid medullary carcinoma.

a *χ*^2^ test.
